# Supplementary material for: Inconsistency in Shoulder Arthrometers for Measuring Glenohumeral Joint Laxity: A Systematic Review
Source: Bioengineering (Basel). 2023 Jul 4;10(7):799. doi: 10.3390/bioengineering10070799 (PMC10376824; doi:10.3390/bioengineering10070799)
Supplement: Supplementary file 1 [file bioengineering-10-00799-s001.zip › bioengineering-2459106-supplementary.pdf]

Table S1. Database search strategy.

Database	Results	Search strategy	Date
**PubMed**	667	(shoulder OR glenohumeral OR glenoid) AND (arthrometer OR telos OR KT1000 OR KT2000 OR donjoi OR laxometer OR laxity)	03/03/2023
**EMBASE**	1149	('shoulder'/exp OR shoulder OR glenohumeral OR 'glenoid'/exp OR glenoid) AND ('arthrometer'/exp OR arthrometer OR 'telos'/exp OR telos OR kt1000 OR kt2000 OR donjoi OR laxometer OR laxity)	03/03/2023
**Web of Science**	798	(shoulder OR glenohumeral OR glenoid) AND (arthrometer OR telos OR KT1000 OR KT2000 OR donjoi OR laxometer OR laxity) (All Fields)"	03/03/2023
**Total**	**2614**		

Table S2. Risk of bias criteria defined according to Risk of Bias Assessment tool for Non-randomized Studies (RoBANS) tool.

Domain	Description
**Selection of participants**	**Selection bias** caused by inadequate selection of participants.<br><br>Selection of individuals (regardless of the level of physical activity) with or without symptomatic shoulders.<br><br>Selection of healthy individuals (athletes or non-athletes) should comprise of those with asymptomatic shoulders and without history of any known shoulder injury or shoulder symptoms (e.g., pain or disability).<br><br>Selection of injured individuals (athletes or non-athletes) should comprise of those with symptomatic shoulders, with a known and diagnosed injured that can affect shoulder joint laxity, including history of shoulder dislocation (either traumatic or non-traumatic) and complaints of shoulder instability (anterior, posterior, inferior or multidirectional). Other shoulder injuries may be considered, but not compulsory, such as labral tears, Bankart or Hill-Sachs lesions. Selection of injured individuals could also be made by enrolling a population that was submitted to shoulder stabilization surgery.
**Confounding variables**	**Selection bias** caused by inadequate confirmation and consideration of confounding variables.<br><br>As confounding variables, we consider: level of sports participation (athletes vs non-athletes); age differences; sex discrepancies (males vs females); and shoulder dominance. When including a single group, the age and sex intra-group discrepancies are not considered. If including a control group with the contralateral shoulder, this control group should be comprised by a homogenous and comparable sample.
**Exposure measurement**	**Performance bias** caused by inadequate measurement of exposure.<br><br>Laxity measurement should be made through the calculation of shoulder bone displacement (usually combined imaging assessment) to measure the precise joint laxity. Shoulder positioning (rotation and abduction) should be reported or shown to allow comparable results.<br><br>Stiffness measurement should be made through the computation of force-displacement curves. To calculate stiffness, the arthrometer must allow the application of progressive (controlled) load and record the shoulder movement for each load increment. An imaging control would be preferable to measure stiffness, but it was not be compulsory.
**Blinding outcome assessment**	**Detection bias** caused by inadequate blinding of outcome assessment.<br><br>Evaluator and/or data analysist not blinded to group or participant conditioning during the exam with the arthrometer or when making the measurement in the imaging exams.
**Incomplete outcome data**	**Attrition bias** caused by inadequate handing of incomplete data outcome.<br><br>Missing data or loss to follow-up in >5% of outcome variables.

**Selective outcome reporting**	**Reporting bias** caused by selective outcome reporting.<br><br>Because included studies are not traditional clinical trials, an a priori protocol registry is not expected. Reporting bias will be judged based on the reporting of the collected/assessed outcomes at methods and results.

Table S3. Population characteristics of included studies.

Reference	Country	K (population)	N (shoulders)	Sex (M/F)	Age, M ± SD [range], yrs	Weight, M±SD (Kg)	Height, M±SD (cm)	Characteristics of population
Azarsa et al. (2021) [19]	Iran	20	20	20/0	37 ± 7.47 [27-55]	81.35 ± 6.99	175.25 ± 0.08	Volunteer individuals (asymptomatic shoulders).
Borsa et al. (1999) [20]	USA	20	40	10/10	25.1 ± 5.5	NR	NR	Physically active volunteer individuals (asymptomatic shoulders).
Borsa et al. (2000) [21]	USA	51	102	24/27	Male: 22.1 ± 2.5<br>Female: 22.4 ± 3.1	Male: 78.7 ± 11.1<br>Female: 63.0 ± 7.5	Male: 177.7 ± 9.6<br>Female: 165.7 ± 8.0	Physically active volunteer individuals (asymptomatic shoulders).
Borsa et al. (2001,2002) [22, 23]	USA	20	20	9/11	20.9 ± 3.6	NR	NR	Volunteer individuals (asymptomatic shoulders).
Borsa et al. (2005)a [24]	USA	43	86	NR	25.1 ± 3.3	96.8 ± 10.1	188.2 ± 6.1	Professional baseball pitchers (major and minor league pitchers) with asymptomatic shoulder and no history of surgery
Borsa et al. (2005)b* [25]	USA	Experiment 1: 20<br><br>Experiment 2: 13	Experiment 1: 20<br><br>Experiment 2: 13	Experiment 1: 11/9<br><br>Experiment 2: 10/3	Experiment 1: 22.9 ± 3.3<br><br>Experiment 2: 22.9 ± 3.4	Experiment 1: 70.7 ± 11.5<br><br>Experiment 2: 75.2 ± 15.5	Experiment 1: 172.6 ± 12.3<br><br>Experiment 2: 175.5 ± 14.2	Healthy individuals with asymptomatic shoulder
Borsa et al. (2005)c [26]	USA	Group I: 42<br><br>Group II: 44	Group I: 84<br><br>Group II: 88	Group I: 26/16<br><br>Group II: 26/18	Group I: 19.4 ± 1.6 (male); 19.7 ± 1.0 (female)	Group I: 82.3 ± 6.2 (male); 65.5 ± 4.5 (female)	Group I: 187.9 ± 6.6 (male); 170 ± 7.2 (female)	Group I: NCAA Division I

					Group II: 21.5 ± 3.3 (male); 18.7 ± 0.6 (female)	Group II: 79.2 ± 16.6 (male); 62.3 ± 8.5 (female)	Group II: 179.4 ± 9.7 (male); 165.3 ± 5.5 (female)	Swimmers with no history of shoulder pain (asymptomatic shoulders) and with a history of shoulder pain<br><br>Group II: Healthy individuals without history of long-term participation (>5 years) in overhead sports or occupations (asymptomatic shoulders)
Borsa et al. (2006) [27]	USA	34	68	NR	24.4 ± 3.7	94.5 ± 9.6	188.3 ± 6.2	Professional baseball pitchers (asymptomatic shoulders)
Crawford & Sauers (2006) [28]	USA	22	44	NR	16.50 ± 0.74	75.43 ± 13.24	178.51 ± 7.66	High school baseball pitchers (asymptomatic shoulders)
Ellenbecker et al. (2000) [29]	USA	20	40	NR	21.25 ± 2.31 [18 - 30]	NR	NR	Professional baseball pitchers (asymptomatic shoulders)
Friscia et al. (2008) [30]	USA	Group I: 15<br><br>Group II: 15	Group I: 30<br><br>Group II: 30	Group I: 15/0<br><br>Group II: 15/0	Group I: 16.1 ± 1.2<br><br>Group II: 17.3 ± 1.0	Group I: 72.9 ± 16.9	Group I: 174.9 ± 6.9	Group I: High school baseball athletes with ≥2 consecutive years of

						Group II: 79.9 ± 12.8	Group II: 183.5 ± 8.4	participation (asymptomatic shoulders).<br><br>Group II: Individuals that did not participate in sports throwing in the past five years (asymptomatic shoulders).
Hatzel et al. (2006) [31]	USA	46	46	19/27	22.47 ± 3.5	70.1 ± 11.7	165.2 ± 9.1	Healthy individuals (asymptomatic shoulder).
Jørgensen & Bak (1995) [32]	Denmar k	Group I: 10<br><br>Group II: 10<br><br>Group III: 10<br><br>Group IV: 10<br><br>Reproducibil ity group: 20	Group I: 20<br><br>Group II: 20<br><br>Group III: 20<br><br>Group IV: 20<br><br>Reproducibil ity group: 40	Group I: 5/5<br><br>Group II: 7/3<br><br>Group III: 5/5<br><br>Group IV: 1/9<br><br>Reproducibilit y group: NR	Group I: Median age 25 [15 - 40]<br><br>Group II: Median age 30 [18 - 49]<br><br>Group III: Median age 23 [16-45]<br><br>Group IV: Median age 24 [18-45]<br><br>Reproducibility group: Median age 27 [15-49]	NR	NR	Group I: Individuals with bilateral healthy shoulders.<br><br>Group II: Individuals with history of a unilateral, traumatic anterior shoulder dislocation<br><br>Group III: Overhead athletes without history of traumatic dislocation, but with unilateral pain and a sensation of instability during overhead activity

								Group IV: Individuals with atraumatic multidirectional instability due to generalized joint hyperlaxity<br><br>Reproducibility group: Individuals among the hospital staff (asymptomatic shoulder) and patients seen with known instability
Jørgensen et al. (1999) [33]	Denmar k	Group I: 21<br><br>Group II: 20	Group I: 21<br><br>Group II: 20	Group I: 15/6<br><br>Group II: 15/5	Group I: 28 [20–41]<br><br>Group II: 28 [18–51]	NR	NR	Group I: Individuals that had arthroscopic surgery for recurrent post-traumatic anterior shoulder dislocation<br><br>Group II: Individuals that had open surgery for recurrent post-traumatic anterior shoulder dislocation
Laudner et al. (2012)a [34]	USA	58	116	58/0	22.6 ± 2.6	88.2 ± 8.2	188.7 ± 4.6	Professional baseball pitchers (asymptomatic shoulders).

Laudner et al. (2012)b [35]	USA	30	30	30/0	20.2 ± 1.4	88.9 ± 9.7	185.0 ± 5.0	Collegiate baseball players - pitchers and position players (asymptomatic shoulders).
Laudner et al. (2013) [36]	USA	Group I: 24<br><br>Group II: 17	Group I: 24<br><br>Group II: 17	Group I: 0/24<br><br>Group II: 0/17	Group I: 19.6 ± 1.8<br><br>Group II: 18.8 ± 0.9	Group I: 60.7 ± 16.3<br><br>Group II: 56.1 ± 6.2	Group I: 163.8 ± 9.9<br><br>Group II: 161.3 ± 4.7	Group I: cheerleaders of varsity team (asymptomatic shoulders).<br><br>Group II: cheerleaders of competition team (asymptomatic shoulders).
Park et al. (2016) [37]	Korea	Group I: 36<br><br>Group II: 23	Group I: 36<br><br>Group II: 46	Group I: 28/8<br><br>Group II: 19/4	Group I: Median age 26.1 [15-57]<br><br>Group II: Median age 19.7 [14-25]	NR	NR	Group I: Individuals who experienced anterior shoulder dislocation (27 individuals with anterior instability and 9 with multidirectional instability)<br><br>Group II: Volunteers without abnormal symptoms.
Pizzari et al. (1999) [38]	Australia	28	56	12/16	22.1 ± 2.9 [19-34]	NR	NR	Undergraduate physiotherapy students (asymptomatic shoulders).

Sauers et al. (2001)a [39]	USA	20	40	13/12	21.9 ± 2.6	NR	NR	Healthy individuals (asymptomatic shoulder).
Sauers et al. (2001)b [40]	USA	51	102	23/28	22 ± 2.8	NR	NR	Recreational athletes (asymptomatic shoulder).
Taylor & Bandy (2005) [41]	USA	15	15	6/9	25 ± 4	NR	NR	Volunteer individuals (asymptomatic shoulders).

M: male; F: Female; yrs: Years; Kg: kilograms; cm centimetres; M: Mean; SD: standard deviation; NR: not reported

* Experiment 1 – to quantify glenohumeral laxity in asymptomatic shoulders using a graded stress technique; Experiment 2- to determine test-retest and interrater repeatability of the sonographic imaging method and measurement procedure

Table S4. Device characteristics and measurement of GH laxity/stiffness.

Reference	Device	Method of force application	Amount of load	Direction of force	Shoulder position	Shoulder fixation	Laxity measurement system	Procedure
Azarsa et al. (2021) [19]	Custom-designed robotic device	E-MECH	10 - 80 N	Inferior	Individuals were placed in a supine position. Shoulder at 90°of abduction and external rotation.	Gripper platform for upper limb positioning and fixation.	Digital motion controller + Visual Studio C++ software	Mean of the slopes of the linear portions of the force – displacement curves as the independent variables (stiffness)
Borsa et al. (1999) [20]	Customized instrumented shoulder arthrometer	H-MECH	67, 89, 111 and 134 N	PA and AP	Individuals were placed in a sitting position. Shoulder at 20° abduction and neutral rotation, with the elbow at 90° flexion and forearm pronated.	Crossed torso straps (anterior fixation) and another strap at the elbow (anterior fixation). The experimenter additionally stabilized the scapula with his thumb (coracoid process) and index finger (scapular spine) during each trial.	Sensor: records the shoulder position (acromion and humeral head).	Amount of displacement of the humeral head and acromion.
Borsa et al. (2000) [21]	Customized instrumented shoulder arthrometer	H-MECH	0 - 134 N	PA and AP	Individuals were placed in a sitting position. Shoulder at 20° abduction and neutral rotation, with the elbow at 90°	Crossed torso straps (anterior fixation) and another strap at the elbow (anterior fixation). The experimenter additionally stabilized the scapula with his	Sensor: records the shoulder position	Amount of displacement of the humeral head and acromion.

					flexion and forearm pronated.	thumb (coracoid process) and index finger (scapular spine) during each trial.		
Borsa et al. (2001, 2002) [22, 23]	Customized instrumented shoulder arthrometer	H-MECH	NR (until a capsular endpoint)	PA, AP and inferior	Individuals were placed in a sitting position. Shoulder at 20° abduction and neutral rotation, with the elbow at 90° flexion and forearm pronated.	Crossed torso straps (anterior fixation) and another strap at the elbow (anterior fixation). The experimenter additionally stabilized the scapula with his thumb (coracoid process) and index finger (scapular spine) during each trial. For inferior translation, the elbow strap is removed.	Sensor: records the shoulder position for sagittal translation (acromion and humeral head) and inferior translation (humeral head and lateral epicondyle of the distal humerus).	Amount of displacement of the humeral head and acromion; and for inferior translation, the amount of displacement of the humeral head and lateral epicondyle of the distal humerus.
Borsa et al. (2005)a [24]	TELOS (Telos, Weiterstadt, Germany)	H-MECH	15 daN (150 N)	PA and AP	Individuals were placed in a sitting position. Shoulder at 90° of abduction in the scapular plane and 60° of external rotation.	Two adjustable counterbearings for stabilizing the shoulder girdle (scapula and coracoid process).	Portable US scanner.	Distance between the posterior glenoid to the posterior humeral head along the scapular plane.
Borsa et al. (2005)b* [25]	TELOS (Telos, Weiterstadt, Germany)	H-MECH	Experiment 1: 10 daN (100 N)	PA and AP	Individuals were placed in a sitting position. Shoulder at 90° of abduction in the	Two adjustable counterbearings for stabilizing the shoulder girdle (scapula and coracoid process).	Standard radiography and portable US scanner.	Radiography: distance between the center of the humeral head from the center of the glenoid.

			Experiment 2: 0 -10 daN (100 N)		scapular plane and 60° of external rotation.			US: distance in glenohumeral position between the baseline image (0-dN force) and stressed image (10-dN force).
Borsa et al. (2005)c [26]	TELOS (Telos, Weiterstadt, Germany)	H-MECH	15 daN (150 N)	PA and AP	Individuals were placed in a sitting position. Shoulder at 90° of abduction in the scapular plane and 60° of external rotation.	Two adjustable counterbearings for stabilizing the shoulder girdle (scapula and coracoid process).	Portable US scanner.	Distance between the posterior glenoid to the posterior humeral head along the scapular plane.
Borsa et al. (2006) [27]	TELOS (Medical, Austin & Associates, Inc., Fallston, MD) + LigMaster™ (Sport Tech, Inc., Charlottesville, VA)	H-MECH	15 daN (150 N)	PA and AP	Individuals were placed in a sitting position. Shoulder at 90° of abduction in the scapular plane and 60° of external rotation.	Two adjustable counterbearings for stabilizing the shoulder girdle (scapula and coracoid process).	Sensor: LigMaster™ system.	Algorithm that determines the best fit of the two lines by minimization of mean squared error to calculate the slope force-displacement curve (stiffness).
Crawford & Sauers (2006) [28]	Modified Telos GA—II/E + LigMaster™, (Sports Tech,	H-MECH	15 daN (150 N)	PA and AP	Individuals were placed in a sitting position. Anterior GH laxity: shoulder in 90° of	Coracoid process and anterior proximal forearm.	Sensor: LigMaster™ system.	Algorithm that determines the best fit of the two lines by minimization of mean squared error to

	Charlottesvil l, VA)				abduction at neutral rotation and then at 90° of external rotation.<br><br>Posterior GH laxity: Individuals were placed in a sitting position. shoulder in 90° of abduction and neutral rotation.			calculate the slope force-displacement curve (stiffness).<br><br>Displacement between the displacement data point nearest the calculated inflection point and the terminal displacement point recorded at 15 daN of force (laxity).
Ellenbecker et al. (2000) [29]	Telos (Gendex 5000, Universal Inc., Chicago, Illinois)	H-MECH	15 daN (150 N)	PA	Individuals were placed in a sitting position. Shoulder in 90° of abduction at the scapular plane tested with neutral rotation and then at 60° of external rotation.	Two adjustable counterbearings for stabilizing the shoulder girdle (scapula and coracoid process).	Standard radiography.	Distance between the center of the humeral head from the center of the glenoid.
Friscia et al. (2008) [30]	Modified Telos GA—II/E + Ligmaster™ (version 1.10, Sport Tech Inc., Charlottesvil le, VA)	H-MECH	15 daN (150 N)	PA	Individuals were placed in a sitting position. Shoulder with 90° abduction, 90° external rotation and 90° of elbow flexion.	Coracoid process and anterior proximal forearm.	Sensor: LigMaster™ system.	GH laxity is calculated as the total amount of displacement point recorded at 15 daN of force.

Hatzel et al. (2006) [31]	Modified KT-2000 (MEDmetric Corporation, San Diego, CA)	H-MECH	67 N<br>89 N<br>134 N	PA	Individuals were placed in a supine position. Shoulder at 90° abduction, 45° of horizontal adduction, and neutral rotation.	Sterno-clavicular area (anteriorly by the KT-2000) and scapulothoracic articulation (by the rigid extension from the device).	Sensor: records the amount of translation of the device.	Amount of displacement occurring at the GH joint in the sagittal plane.
Jørgensen & Bak (1995) [32]	Donjoy® Laxity Tester (Smith and Nephew Donjoy)	H-MECH	20Ibs (89 N)	PA and AP global	Individuals were placed in a sitting position. Shoulder at 20° abduction and in neutral rotation and elbow flexed at 90°.	Scapula is fixed by examinator's thumb and index finger at the acromion.	Visual-instrumented: scale on the spring balance at intervals of 0.5 mm.	Amount of displacement occurring at the GH joint in the sagittal plane.
Jørgensen et al. (1999) [33]	Donjoy® Laxity Tester (Smith and Nephew Donjoy)	H-MECH	20 lbs (89 N)	PA and AP global	Individuals were placed in a sitting position. Shoulder at 20° abduction and in neutral rotation and elbow flexed at 90°.	Scapula is fixed by examinator's thumb and index finger at the acromion.	Visual-instrumented: scale on the spring balance at intervals of 0.5 mm.	Amount of displacement occurring at the GH joint in the sagittal plane.
Laudner et al. (2012)a [34]	Modified Telos GA—II/E + LigMaster™, (Sports Tech, Charlottesvil l, VA)	H-MECH	12 daN (120 N)	PA	Individuals were placed in a sitting position. Shoulder in 90° of abduction and 90° of external rotation, while the elbow was in 90°	Coracoid process and anterior proximal forearm.	Sensor: LigMaster™ system.	Laxity (mm): difference in displacement between the inflection point (at the end of soft tissue compression and the initiation of humeral

					of flexion and full pronation.			head translation) and the final amount of displacement recorded at 12 daN of anterior force.
Laudner et al. (2012)b [35]	Modified Telos GA—II/E + LigMaster™, (Sports Tech, Charlottesvil l, VA)	H-MECH	12 daN (120 N)	PA	Individuals were placed in a sitting position. Shoulder in 90° of abduction and 90° of external rotation, while the elbow was in 90° of flexion and full pronation.	Coracoid process and anterior proximal forearm.	Sensor: LigMaster™ system.	Laxity (mm): difference in displacement between the inflection point (at the end of soft tissue compression and the initiation of humeral head translation) and the final amount of displacement recorded at 12 daN of anterior force.
Laudner et al. (2013) [36]	Modified Telos GA—II/E + LigMaster™, (Sports Tech, Charlottesvil l, VA)	H-MECH	12 daN (120 N)	PA	Individuals were placed in a sitting position. Shoulder in 90° of abduction and 90° of external rotation, while the elbow was in 90° of flexion and full pronation.	Coracoid process and anterior proximal forearm.	Sensor: LigMaster™ system.	Laxity (mm): difference in displacement between the inflection point (at the end of soft tissue compression and the initiation of humeral head translation) and the final amount of displacement recorded at 12 daN of anterior force.

								Stiffness (N/mm): amount of force between the inflection point and the terminal force (12 daN) divided by the amount of laxity (displacement) (12 daN).
Park et al, (2016) [37]	TELOS (Telos, Weiterstadt, Germany)	H-MECH	15 daN (150 N)	PA and AP	Individuals were placed in a sitting position. Shoulder in 90° of abduction at neutral rotation and then at 60° of external rotation.	Two adjustable counterbearings for stabilizing the shoulder girdle (scapula and coracoid process).	Standard radiography.	Distance between the center of the humeral head from the center of the glenoid.
Pizzari et al. (1999) [38]	KT-1000 Ligament Arthrometer (MEDmetric Corporation, San Diego, California)	H-MECH	67 N	AP	Individuals were placed in a supine position. Shoulder in 90° of abduction and 90° of external rotation, with the arm resting on the plinth.	Velcro straps around the arm to stabilize with the arthrometer; shoulder girdle stabilized with pressure exerted by the tester's hand over the scapula and the distal sensor pad of the KT-1000.	Sensor: records the amount of translation of the device.	Amount of displacement occurring at the GH joint in the sagittal plane.
Sauers et al. (2001)a [39]	Customized instrumented shoulder arthrometer	H-MECH	67, 89, 111, and 134 N	PA and AP	Individuals were placed in a sitting position. Shoulder at 20° abduction and neutral	Crossed torso straps (anterior fixation) and another strap at the elbow (anterior fixation). The	Sensor: records the shoulder position (acromion and humeral head).	Amount of displacement of the humeral head and acromion.

					rotation, with the elbow at 90° flexion and forearm pronated.	experimenter additionally stabilized the scapula with his thumb (coracoid process) and index finger (scapular spine) during each trial.		
Sauers et al. (2001)b [40]	Customized instrumented shoulder arthrometer	H-MECH	67, 89, 111, and 134 N	PA and AP	Individuals were placed in a sitting position. Shoulder at 20° abduction and neutral rotation, with the elbow at 90° flexion and forearm pronated.	Crossed torso straps (anterior fixation) and another strap at the elbow (anterior fixation). The experimenter additionally stabilized the scapula with his thumb (coracoid process) and index finger (scapular spine) during each trial.	Sensor: records the shoulder position (acromion and humeral head).	Amount of displacement of the humeral head and acromion.
Taylor & Bandy (2005) [41]	KT-1000 Ligament Arthrometer (MEDmetric Corporation, San Diego, California)	H-MECH	67 N	PA	Position 1: Individuals were placed in a supine position. Shoulder abducted to 20° and externally rotated to 0° with the arm resting on the plinth.<br><br>Position 2: Individuals were	Velcro straps around the arm to stabilize with the arthrometer; shoulder girdle stabilized with pressure exerted by the tester's hand over the scapula and the distal sensor pad of the KT-1000.	Sensor: records the amount of translation of the device.	Amount of displacement occurring at the GH joint in the sagittal plane.

					placed in a supine position. Shoulder in 90° of abduction and 90° of external rotation, with the arm resting on the plinth.			

NR: not reported; GH: Glenohumeral; US: ultrasound; AP: anterior-posterior; PA: posterior-anterior; AER: anterior drawer stress radiograph with 0° external rotation; PER0: posterior drawer stress radiography with 0° external rotation; AER0: anterior drawer stress radiograph with 60° external rotation; PER60: posterior drawer stress radiography with 60° external rotation, PCC: Pearson correlation coefficient; H-MECH*:* hand-held mechanical force application, with force measurement (e.g., by load cells**);** E-MECH: electromechanical system, operated by a force–displacement sensor/transducer

* Experiment 1 – to quantify glenohumeral laxity in asymptomatic shoulders using a graded stress technique; Experiment 2- to determine test-retest and interrater repeatability of the sonographic imaging method and measurement procedure

Table S5. Device validity/reliability and laxity/stiffness outcomes.

Reference	Device	Validity	Reliability	Laxity (mm) and stiffness (N/mm)	
Azarsa et al. (2021) [19]	Custom-designed robotized device	NR	ICC [within-session] = 0.96 (0.93–0.98), SEM = 0.08 N/mm<br>ICC [between-session] = 0.97 (0.95–0.99), SEM = 0.07 N/mm<br><br>Repeated measures ANOVAs:<br>Session I: (F [3, 76] = 0.18, p = 0.908)<br>Session II: (F [3, 76] = 0.47, p = 0.70)	Inferior	*Session I:* 1.50 ± 0.40 N/mm<br>*Session II:* 1.52 ± 0.40 N/mm
Borsa et al. (1999) [20]	Customized instrumented shoulder arthrometer	NR	Direction and load ICC = 0.94 (0.90 - 0.97), SEM = 0.88 mm.	PA	*67 N:* 6.1 ± 1.7 mm<br>*89 N:* 7.4 ± 1.8 mm<br>*111 N:* 8.7 ± 1.9 mm<br>*134 N:* 9.7 ± 2.0 mm
				AP	*67 N:* 5.0 ± 2.7 mm<br>*89 N:* 6.1 ± 3.0 mm<br>*111 N:* 6.8 ± 3.7mm<br>*134 N:* 6.5 ± 4.1mm
Borsa et al. (2000) [21]	Customized instrumented shoulder arthrometer	NR	NR	PA	*Men:* 8.3 ± 2.2 mm<br>*Women:* 11.4 ± 2.8 mm<br><br>*Men:* 20.5 ± 5.0 N/mm<br>*Women:* 16.3 ± 4.2 N/mm $^{-1}$
Borsa et al. (2001, 2002) [22, 23]	Customized instrumented shoulder arthrometer	NR	Anterior translation: ICC [2,1] = 0.98<br>Posterior translation: ICC [2,1] = 0.96<br>Inferior translation: ICC [2,1] = 0.98	PA	14.5 ± 2.3 mm<br>16.7 ± 4.5 N/mm
				AP	14.0 ± 2.8 mm<br>15.4 ± 3.5 N/mm
				Inferior	13.9 ± 4.6 mm<br>15.7 ± 5.6 N/mm

Borsa et al. (2005)a** [24]	TELOS (Telos, Weiterstadt, Germany)	NR	NR	PA	*Throwing Arm:* 2.62 ± 1.78 mm<br><br>*Nonthrowing Arm:* 2.99 ± 2.54 mm<br><br>*Difference between throwing and non-Nonthrowing arms:* 0.31 ± 2.94 mm
				AP	*Throwing Arm:* 5.94 ± 3.78 mm<br><br>*Nonthrowing Arm:* 4.82 ± 3.37 mm<br><br>*Difference between throwing and non-Nonthrowing arms:* 1.12 ± 4.67 mm
				Global translation	*Throwing Arm:* 8.72 ± 5.01 mm<br><br>*Nonthrowing Arm:* 7.81 ± 4.79 mm<br><br>*Difference between throwing and non-Nonthrowing arms):* 0.75 ± 5.59 mm
Borsa et al. (2005)b* [25]	TELOS (Telos, Weiterstadt, Germany)	Pearson correlation $r = 0.79$ ($r^2 = 0.62$) between radiography and US.	Intra-rater: ICC = 0.72, SEM = 1.51 mm (PA); ICC = 0.85, SEM = 0.83 mm (AP) Inter-rater: ICC = 0.96, SEM = 0.40 mm (PA); ICC = 0.99, SEM = 0.34 mm (AP)	PA	*Experiment 2 (US):* 2.77 ± 1.4 mm (session 1), 2.97 ± 1.8 mm (session 2), 2.39 ± 1.85 mm (examiner 1) and 2.19 ± 1.78 mm (examiner 2)
				AP	*Experiment 1:* 3.97 ± 2.2 mm (US) and 2.96 ± 2.0 mm (radiography)<br><br>*Experiment 2 (US):* 5.47 ± 2.5 mm (session 1), 5.03 ± 1.8 mm (session 2), 4.87 ± 3.41 (examiner 1) and 4.89 ± 3.41 mm (examiner 2)

Borsa et al. (2005)c [26]	TELOS (Telos, Weiterstadt, Germany)	NR	NR	PA	*Group I:* 2.74 ± 1.8 mm (no history pain) and 2.90 ± 1,6 (history pain)<br><br>*Group II:* 2.74 ± 1.7 mm<br><br>*Difference between Group I and Group II:* 0.08 ± 1.7 mm
				AP	*Group I:* 5.14 ± 2.6 mm (no history pain and 5.42 ± 2.3 (history pain)<br><br>*Group II:* 4.90 ± 2.7 mm<br><br>*Difference between Group I and Group II:* 0.40 ± 2.5 mm
Borsa et al. (2006) [27]	TELOS (Medical, Austin & Associates, Inc., Fallston, MD) + LigMaster™ (Sport Tech, Inc., Charlottesville, VA)	NR	Right Arms - PA: ICC = 0.29, SEM = 1.17 $N.mm^{-1}$<br>Right Arms-AP: ICC = 0.69, SEM = 1.78 $N.mm^{-1}$<br>Left Arms PA: ICC = 0.49, SEM = 1.28 $N.mm^{-1}$<br>Left Arms - AP: ICC = 0.89, SEM = 1.18 $N.mm^{-1}$	PA	*Throwing Arm:* 16.6 ± 1.9 N/mm<br><br>*Nonthrowing Arm:* 16.2 ± 1.7 N/mm<br><br>*Difference between Throwing and non-Nonthrowing arms:* 0.4 ± 1.8 N/mm^{-1}
				AP	*Throwing Arm:* 15.1 ± 3.4 N/mm<br><br>*Nonthrowing Arm:* 15.3 ± 3.8 N/mm<br><br>*Difference between Throwing and non-Nonthrowing arms:* - 0.2 ± 3.6N/mm^{-1}
Crawford & Sauers (2006)** [28]	LigMaster, (Sports Tech, Charlottesvill, VA)	NR	Laxity:<br>ICC [2,1] = 0.84, SEM = 0.53 mm; ICC [2,k] = 0.83, SEM = 0.43 mm<br><br>Stiffness:	PA	*Throwing Arm:*<br>*Neutral rotation:* 11.90 ± 1.93 mm and 8.05 ± 1.09 N/mm<br>*External rotation:* 9.15 ± 0.91 mm and 10.87 ± 0.98 N/mm

			ICC [2,1] = 0.84, SEM = 0.52 N/mm;<br>ICC [2,k] = 0.88, SEM = 0.37 N/mm		*Nonthrowing Arm:*<br>*Neutral rotation:* 12.45 ± 1.97 mm and 7.77 ± 0.94 N/mm<br>*External rotation:* 9.63 ± 1.24 mm and 10.24 ± 1.19 N/mm
				AP	*Throwing Arm:*<br>*Neutral rotation:* 12.71 ± 2.51 mm and 8.00 ± 1.21 N/mm<br><br>*Nonthrowing Arm:*<br>*Neutral rotation:* 12.46 ± 1.95 mm and 8.05 ± 1.29 N/mm
				Global translatio n	*Throwing Arm:* 24.61 ± 3.67 mm<br><br>*Nonthrowing Arm*: 24.92 ± 3.08 mm
Ellenbecker et al. (2000) [29]	Telos (Gendex 5000, Universal Inc., Chicago, Illinois)	*Pearson correlation between stress radiography and manual translation.*<br><br>Neutral rotation<br>Dominant Arm: r = 0.1194<br>Nondominant Arm: r = 0.3253<br><br>External rotation<br>Dominant Arm: r = 0.0270<br>Nondominant Arm: r = 0.3088	ICC = 0.635 (neutral rotation)<br>ICC = 0.679 (external rotation)	PA	*Dominant Arm:* 2.08 ± 2.97 mm (Neutral rotation) and 1.40 ± 2.14 mm (External rotation)<br><br>*Nondominant Arm:* 2.23 ± 2.41 mm (Neutral rotation) and 2.07 ± 2.10 mm (External rotation)<br><br>*Difference between Dominant and non-Nondominant arm):* - 0.15 ± 4.06 mm (Neutral rotation) and - 0.67 ± 2.93 mm (External rotation)
Friscia et al. (2008) [30]	Modified Telos GA—II/E +	NR	NR	PA	*Baseball Players:* 9.64 ± 1.70 mm 8 dominant Arm) and 9.01 ± 1.33 mm (nondominant Arm)

	Ligmaster™ (version 1.10, Sport Tech Inc., Charlottesville, VA)				*Non-Baseball Players:* 10.89 ± 1.56 mm (dominant Arm) and 10.40 ± 1.02 mm (nondominant Arm)
Hatzel et al. (2006) [31]	KT-2000 knee ligament arthrometer (MEDmetric Corporation, San Diego, CA)	NR	67 N: ICC = 0.887 (95% CI 0.83–0.93), SEM = 0.845 mm<br>89 N: ICC = 0.88 (95% CI 0.81– 0.92), SEM = 0.814 mm<br>134N: ICC = 0.91 (95% CI 0.87– 0.94), SEM = 0.869 mm	PA	*67 N:* 12.55 ± 2.49 (day 1) and 12.35 ± 2.54 (day 2)<br>*89 N:* 17.70 ± 3.09 mm (day 1) and 17.77 ± 3.01 mm (day 2)<br>*134 N:* 27.41 ± 4.37 mm (day 1) and 28.03 ± 3.96 mm (day 2)
Jørgensen & Bak (1995)** [32]	Donjoy® Knee	NR	ICC = 0.996	Global translation	*Group I:* 2.1 ± 1.7 mm (left) and 2.1 ± 1.7 mm (right) and 0.6 ± 0.5 mm (difference between left and right)<br><br>*Group II:* 6.4 ± 3.6 mm (left) and 2.8 ± 2.9 mm (right) and 3.6 ± 2.0 mm (difference between left and right)<br><br>*Group III:* 5.8 ± 26 mm (left) and 3.2 ± 2.0 mm (right) and 2.6 ± 1.8 mm (difference between left and right)<br><br>*Group IV:* 11.9 ± 6.3 mm (left) and 11.0 ± 6.4 mm (right) and 2.3 ± 2.1 mm (difference between left and right)

					Reproducibility group: 3.02 ± 2.24 mm (test) and 3.30 ± 2.27 mm (re-test)
Jørgensen et al. (1999)** [33]	Laxity Tester (Smith and Nephew Donjoy)	NR	NR	Global translation	*Difference between* mean side difference 0.6 ± 2.9 mm (Group I) and 1.1 ± 3.6 mm (Group II)
Laudner et al. (2012)a [34]	Modified Telos GA—II/E + LigMaster, (Sports Tech, Charlottesvill, VA)	NR	NR	PA	10.0 ± 6.2 mm
Laudner et al. (2012)b [35]	Modified Telos GA—II/E + LigMaster, (Sports Tech, Charlottesvill, VA)	NR	NR	PA	14.1 ± 6.0 mm
Laudner et al. (2013) [36]	Modified Telos GA—II/E + LigMaster, (Sports Tech, Charlottesvill, VA)	NR	NR	PA	*Group I:* 13.5 ± 3.1 mm (pre) and 11.6 ± 5.8 mm (post) and 2.0 ± 6.6 mm (difference between pre-test and post-test);<br><br>*Group II:* 11.7 ± 4.1 mm (pre) and 13.5 ± 4.4 mm (post) and -1.8 ± 2.8 mm (difference between pre-test and post-test)

					Group I: 9.4 ± 0.61 N/mm (pre) and 9.4 ± 1.0 N/mm (post) and 0.01 ± 1.0 mm (difference between pre-test and post-test)<br><br>*Group II:* 9.0 ± 0.78 N/mm (pre) and 8.3 ± 1.3 N/mm (post) and 0.8 ± 1.1 mm (difference between pre-test and post-test)
Park et al. (2016) [37]	TELOS (Telos, Weiterstadt, Germany)	*Pearson correlation between anterior drawer test and stress radiography.*<br><br>At AER60 (0.453; p = 0.005); and AER0 (0.529; p = 0.001).<br><br>Under anesthesia and at AER60 (0.287; p =0.264), and AER0 (0.695; p = 0.002).<br><br>*Differences between anterior drawer test and stress radiography (t-test):*<br>At AER60 (p = 0.773) and AER0 (p = 0.529)	NR	PA	*Group I:* 2.0 ± 1.5 mm (AER60) and 3.4 ± 1.3 mm (AER0)<br><br>*Group II:* 0.7 ± 1.1 mm (AER60) and 1.3 ± 1.6 mm (AER0)
				AP	*Group I:* 3.0 ± 4.4 mm (PER60) and 3.6 ± 5.2 mm (PER0)<br><br>*Group II:* 1.3 ± 1.4 mm (PER60) and 1.0 ± 1.2 mm (PER0)
					Difference between Affected Arms X Nonaffected Arms: 1.39 mm (AER60) and 1.97 mm (AER0)
Pizzari et al. (1999) [38]	KT-1000 Ligament Arthrometer (MEDmetric Corporation,	NR	Dominant Arm: ICC = 0.67<br>Nondominant Arm: ICC = 0.76<br><br>Inter-rater reliability: paired t test:<br>Dominant Arm: 0.24, p = 0.81<br>Nondominant Arm: - 0.82, p = 0.42	AP	*Dominant Arm:* 20.2 ± 5.0 mm (test) and 20.1 ± 4.7 mm (retest) and -0.179 ± 3.98 mm (difference between test and retest)<br><br>*Nondominant Arm:* 21.5 ± 4.8 mm (test) and

	San Diego, California)				22.0 ± 4.9 mm (retest) and 0.518 ± 3.36 mm (difference between test and retest)
Sauers et al. (2001)a [39]	Customized instrumented shoulder arthrometer	NR	Between-trial: $ICC_{2,1}$ = 0.92 (0.77–0.96), SEM = 0.56 mm (0.45 – 0.73 mm)<br><br>Between-session (Intra-rater): $ICC_{2,1}$ = 0.73 (0.60–0.88), SEM = 1.5 mm (0.79–1.9 mm)<br><br>Between-examiner (Inter-rater): $ICC_{2,1}$ = 0.74 (0.66–0.81), SEM = 1.7 mm (1.3 – 2.1 mm)	PA	*67 N:* 7.5 ± 2.1 mm<br>*89 N:* 8.9 ± 2.3 mm<br>*111 N:* 10.2 ± 2.6 mm<br>*134 N:* 11.3 ± 2.8 mm
				AP	*67 N:* 9.3 ± 2.2 mm<br>*89 N:* 10.7 ± 2.3 mm<br>*111 N:* 11.8 ± 2.4 mm<br>*134 N:* 12.7 ± 2.5 mm
Sauers et al. (2001)b [40]	Customized instrumented shoulder arthrometer	NR	NR	PA	*67 N:* 8.0 ± 2.2 mm<br>*89 N:* 9.4 ± 2.5 mm<br>*111 N:* 10.7 ± 2.7 mm<br>*134 N:* 11.9 ± 2.9 mm
				AP	*67 N:* 8.6 ± 2.7 mm<br>*89 N:* 9.9 ± 3.0 mm<br>*111 N:* 10.9 ± 3.2 mm<br>*134 N:* 11.8 ± 3.3 mm
Taylor & Bandy (2005) [41]	KT-1000 Ligament Arthrometer (MEDmetric Corporation, San Diego, California)	NR	Position 1: ICC = 0.93 (95% CI 0.81–0.98)<br>Position 2: ICC= 0.93 (95% CI 0.80–0.97)<br><br>Dependent *t-test* (position 1/position 2) = *P* = 0.01	PA	*Position 1:* 11 ± 3 mm (test) and 10 ± 2 mm (retest)<br><br>*Position 2:* 6 ± 2 mm (test) and 6 ± 1 mm retest)

NR: not reported; GH: Glenohumeral; US: ultrasound; AP: anterior-posterior; PA: posterior-anterior; AER: anterior drawer stress radiograph with 0° external rotation; PER0: posterior drawer stress radiography with 0° external rotation; AER0: anterior drawer stress radiograph with 60° external rotation; PER60: posterior drawer stress radiography with 60° external rotation

*Experiment 1 – to quantify glenohumeral laxity in asymptomatic shoulders using a graded stress technique; Experiment 2- to determine test-retest and interrater repeatability of the sonographic imaging method and measurement procedure
**Global translation represents PA + AP

Figure S1. Risk of bias judgement.

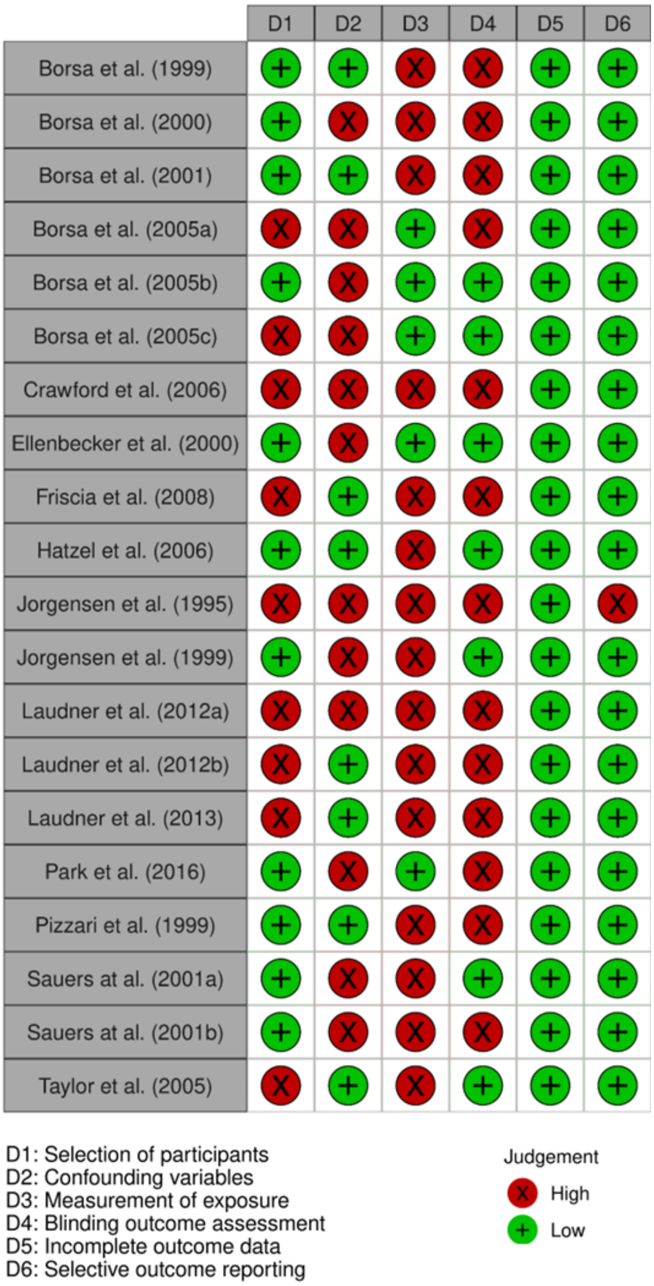

Figure S1.1. Individual study risk of bias for GH laxity.

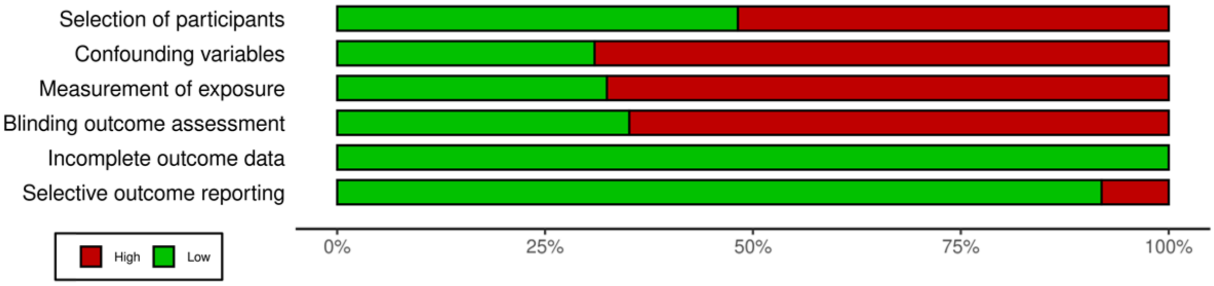

Figure S1.2 – Overall domain-based risk of bias for GH laxity.

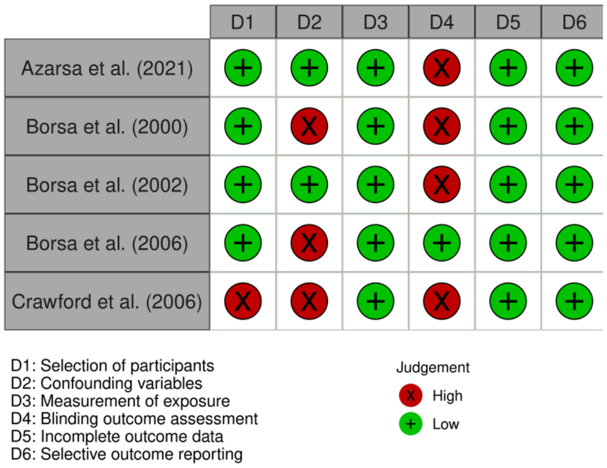

Figure S1.3. Individual study risk of bias for GH stiffness.

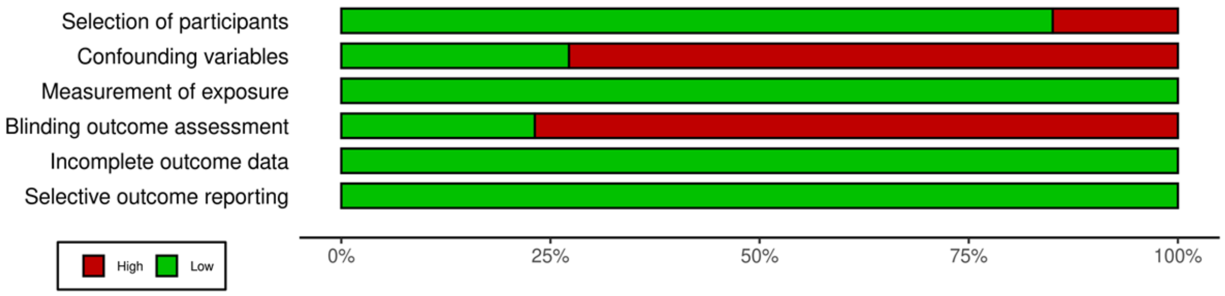

Figure S1.4. Overall domain-based risk of bias for GH stiffness.
